# Live-cell imaging of rapid calcium dynamics using fluorescent, genetically-encoded GCaMP probes with *Aspergillus fumigatus*

**DOI:** 10.1016/j.fgb.2020.103470

**Published:** 2020-09-24

**Authors:** Alberto Muñoz, Margherita Bertuzzi, Constanze Seidel, Darren Thomson, Elaine M. Bignell, Nick D. Read

**Affiliations:** Manchester Fungal Infection Group, Division of Infection, Immunity and Respiratory Medicine, University of Manchester, CTF Building, 46 Grafton Street, Manchester M13 9NT, UK

**Keywords:** *Aspergillus fumigatus*, Calcium imaging, GCaMP calcium probes, live-cell imaging

## Abstract

Calcium signalling plays a fundamental role in fungal intracellular signalling. Previous approaches (fluorescent dyes, bioluminescent aequorin, genetically encoded cameleon probes) with imaging rapid subcellular changes in cytosolic free calcium ([Ca^2+^]_c_) in fungal cells have produced inconsistent results. Recent data obtained with new fluorescent, genetically encoded GCaMP probes, that are very bright, have resolved this problem. Here, exposing conidia or conidial germlings to high external Ca^2+^, as an example of an external stressor, induced very dramatic, rapid and dynamic [Ca^2+^]_c_ changes with localized [Ca^2+^]_c_ transients and waves. Considerable heterogeneity in the timing of Ca^2+^ responses of different spores/germlings within the cell population was observed.

## Introduction

Filamentous fungi need to respond rapidly to the diverse environmental signals that they encounter in their naturally heterogeneous microhabitats. Fast responding intracellular signalling pathways are necessary to quickly activate and regulate downstream elements to elicit appropriate responses to these environmental stimuli. Intracellular Ca^2+^ signalling possesses the necessary properties to fulfil the role for such rapid signalling (Berridge *et al*., 2000, Kudla *et al*., 2010) but its dynamics have been little analysed in fungi at the single-cell level because of the lack of routine means to easily image its often very rapid and dynamic changes.

Calcium signalling and homeostasis are essential for hyphal growth, differentiation and virulence of filamentous fungi (Juvvadi *et al*., 2014), including calcium signalling-dependent multi-drug resistance (Li *et al*., 2019). Indeed, in *Aspergillus nidulans*, transient Ca^2+^ pulses have been demonstrated to coordinate actin assembly and hence stepwise hyphal extension (Takeshita *et al*., 2017). The low resting level (50-100 nM) of cytosolic free Ca^2+^ ([Ca^2+^]_c_) is maintained by Ca^2+^-pumps and -antiporters, and cytoplasmic Ca^2+^-buffering. However, [Ca^2+^]_c_ becomes an intracellular signal when its concentration is transiently increased and this is typically very rapid and commonly localised within a cell (Berridge *et al*., 2000, Kudla *et al*., 2010).

Previous [Ca^2+^]_c_ imaging with Ca^2+^-sensitive dyes produced very variable results with cells often not taking up dye or sequestering dye within organelles (Hickey *et al*., 2004). We developed an easy, routine method for [Ca^2+^]_c_ measurement in filamentous fungi (Nelson *et al*., 2004), including *Aspergillus fumigatus* (Munoz et al., 2015), using 96-well plate luminometry with genetically encoded aequorin as a Ca^2+^-reporter. This bioluminescent probe is well suited for average quantitative measurements of Ca^2+^-signatures in cell populations but not for the imaging/measurement of [Ca^2+^]_c_ dynamics at the single-cell level because its light output is too low and it is biochemically consumed during [Ca^2+^]_c_ measurement (Munoz *et al*., 2015). A newer approach has been to use genetically encoded ratiometric cameleon probes (Kim *et al*., 2012, Kim *et al*., 2015) but issues with low expression of this reporter in some fungi (e.g. *A. fumigatus, Neurospora crassa*, unpubl. results) has limited its widespread exploitation. In the current study we report the use of a newer class of genetically encoded reporters called GCaMPs (Akerboom *et al*., 2012, Chen *et al*., 2013) which have various superior properties for [Ca^2+^]_c_ imaging in filamentous fungi. GCaMP reporters are composed of a circularly permuted (cp) GFP in between the calmodulin (CaM)–interacting region of chicken myosin light chain kinase (M13) at the N-terminus and a vertebrate CaM at the C-terminus. Binding of Ca^2+^ causes the M13 and CaM domains to interact and the resulting conformational change leads to an increase in cpGFP fluorescence (Akerboom *et al*., 2012, Chen *et al*., 2013).

## Results and Discussion

The aims of this study were to:

(1) Demonstrate, with a temporal resolution < 1 sec, the very rapid and dynamic subcellular changes in [Ca^2+^]_c_ that can be readily imaged in conidial germlings of *A. fumigatus*.
(2) Determine the subcellular influence of high Ca^2+^ stress (200 mM CaCl_2_) on immediate exposure and prolonged treatment of this high Ca^2+^ stress to conidial germlings.
(3) Determine whether ungerminated conidia undergo Ca^2+^-signalling and can respond to high Ca^2+^ stress.

Previously we have demonstrated that *A. fumigatus* responds to exposure to the following range of physiologically significant stressors: hypo-osmotic and hyper-osmotic shock, oxidative stress, Caspofungin antifungal treatment, and high external Ca^2+^ (200 mM CaCl_2_) stress (Juvvadi *et al*., 2015, Munoz *et al*., 2015). It was previously demonstrated that treatment with 200 mM CaCl_2_ results in transcriptional changes that stabilise 30 min following exposure (Loss *et al*., 2017). Recently we have also provided evidence that external Ca^2+^ increased as a result of cell injury can act as a ‘danger’ or ‘alarm’ signal that activates a putative innate immune system involved in cell regeneration in filamentous fungi (Medina-Castellanos *et al*., 2018).

We decided to treat conidia and conidial germlings of *A. fumigatus* with high external Ca^2+^ (200 mM CaCl_2_) as an example of an external stressor, and because using it produced a highly reproducible Ca^2+^ signature at the cell population level in 96-multiwell plates (Nelson *et al*., 2004, Munoz *et al*., 2015). We tested and compared strains expressing either GCaMP5 (Akerboom *et al*., 2012) or its derivative GCaMP6, an improved and more sensitive version which was generated via mutagenesis at the interface between cpGFP and CaM (Chen *et al*., 2013) (Movies 1-3). Notably, while GCaMP6 was used only once before, its first use having been in the filamentous fungus *Trichoderma atroviride* to evaluate the regeneration process of hyphae in response to danger signals (Medina-Castellanos *et al*., 2018), the present study reports for the first time the use of GCaMP5 in filamentous fungi. The fluorescence from strains expressing GCaMP6 was significantly brighter than that of GCaMP5 and could be even readily observed with the naked eye through the microscope eyepieces following addition of 200 mM CaCl_2_ (data not shown). Nevertheless, excellent results were still obtainable with the GCaMP5 strain using widefield fluorescence optics and a cooled CCD camera (Movie 1).

The initial experiments were performed with a GCaMP5-expressing strain that had germinated in liquid AMM in a microfluidic chamber and had been treated with high external Ca^2+^ (200 mM CaCl_2_) applied by continuous perfusion. Movie 1 shows a representative example of the results obtained with a group of ten conidial germlings; similar variation in the results were obtained with other conidia germlings of similar lengths.

Prior to stimulation with high Ca^2+^, the [Ca^2+^]_c_-dependent fluorescence of the germlings shown in Movie 1 was low with no obvious subcellular increases in [Ca^2+^]_c_ above resting level in any of the germlings. Upon contact with 200 mM CaCl_2_ applied by controlled perfusion through the cell culture chamber, the cells unexpectedly responded at markedly different times. A variety of changes and dynamics in [Ca^2+^]_c_ were observed. Nine out of the ten cells responded to the Ca^2+^stress and a period of 162 sec passed between the first and the ninth cell reacting with transient [Ca^2+^]_c_ increases even though all of the germlings will have been stimulated at the same time (see also the graph in Suppl. Fig 3A). Transient [Ca^2+^]_c_ elevations were observed five times in one of the cells, three in five of the cells, two in two of the cells and one in one of the cells. The [Ca^2+^]_c_ typically increased in either a specific subcellular region or throughout a whole cell, although sometimes [Ca^2+^]_c_ was observed to increase in two subcellular regions at more-or-less the same time. Waves of [Ca^2+^]_c_ developed from the [Ca^2+^]_c_ foci and moved down the lengths of germ tubes. As reported previously in *Magnaporthe oryzae, Fusarium oxysporum*, and *Fusarium graminearum* (Kim et al., 2012), there was no discernible repetitive pattern in [Ca^2+^]_c_ waves along individual fungal hyphae. Furthermore, no co-ordination of the timing of the initiation of [Ca^2+^]_c_ waves was observed between the groups of germlings analysed (Movies 1, 2). However, it was clear that long germlings produced stronger [Ca^2+^]_c_ signals than short ones (Movies 1, 2).

The velocities of the [Ca^2+^]_c_ waves were of a similar magnitude in germ tubes and more mature hyphae. Quantitative measurements of 12 [Ca^2+^]_c_ waves (two in germ tubes and 10 in more mature hyphae) in GCaMP5-expressing cells at ~ 25°C were combined and found to have an average wave velocity of 4.1 ± 2.1 μm/sec, which is within the range reported for animal cells (typically up to about 30 μm/sec) (Jaffe, 1993). Intracellular [Ca^2+^]_c_ waves in animal cells are generated by Ca^2+^-induced Ca^2+^-release (Zhao *et al*., 2001).

We next analysed the influence of prolonged high Ca^2+^ stress (200 mM CaCl_2_) over 20 min on conidial germlings at the end of which an extra 200 mM CaCl_2_ treatment was applied. The group of longer germlings and hyphae that were analysed (Movie 2) were more advanced in growth than in Movie 1. Playing back the movie at a faster frame rate (23.5 times faster) in this movie highlighted that at this later stage of morphogenesis, [Ca^2+^]_c_ waves more commonly arose from the tips than from subapical regions. Bidirectional [Ca^2+^]_c_ waves were common when they originated from localised subapical foci. Again, considerable heterogeneity in the timing of [Ca^2+^]_c_ responses of the 25 different germlings within the cell population was observed. Capturing images at 1.8 sec intervals to generate this movie induced significant photobleaching of the GCaMP6. If required, the photobleaching could have been expeditiously decreased by reducing the excitation intensity or frequency of image capture. The gain setting on the cooled CCD was increased on the detector after ~ 13 min to adjust for this in order that the fluorescence signal roughly equated to that at the beginning of the movie. Precise quantification of the [Ca^2+^]_c_ would require correction for photobleaching (Carbo *et al*., 2017). The perfusion of an extra 200 mM CaCl_2_ resulted in a global increase in [Ca^2+^]_c_ throughout all fungal hyphaes, as result of Ca^2+^ uptake from the external medium (AMM containing 200 mM CaCl_2_). Whether this was Ca^2+^-channel dependent or cell stress dependent would require further analysis.

We finally analysed the effects of high Ca^2+^ stress (200 mM CaCl_2_) on a subpopulation of 45 ungerminated, swollen and unpolarised spores of the GCaMP6-expressing strain that had been incubated for 16 h at 25°C and had undergone isotropic growth prior to the formation of germ tubes (d’Enfert, 1997). Almost all the spores (44/45) responded to the Ca^2+^ stress but they exhibited great heterogeneity in the timing and intensity of their [Ca^2+^]_c_-responses (see also the graph in Suppl. Fig. 3B). Furthermore, 71% of the spores exhibited multiple transient [Ca^2+^]_c_ increases. Over a period of 9 min exposure to 200 mM CaCl_2_ perfused through the slide culture chamber, 12 (27%) exhibited one transient increase in [Ca^2+^]_c_, 26 (58%) spores exhibited two to four transient [Ca^2+^]_c_ increases whilst six (13%) exhibited five or more.

In conclusion we have shown continuous, localised, rapid and dynamic changes in [Ca^2+^]_c_ in subcellular regions of *A. fumigatus* germlings in response to the application of high Ca^2+^ stress using GCaMP probes that have a very good signal:noise ratio. Our results demonstrate the extraordinary otherwise-hidden heterogeneity in the responses of different but similar-looking, and apparently developmentally equivalent, spores/germlings, in the cell population. These [Ca^2+^]_c_ changes can be observed in long time-courses (< 15 min) under very controlled conditions with drug and other treatments applied in a continuous flow slide culture system. Short germlings, hyphae and ungerminated spores responded to both immediate and prolonged high Ca^2+^ stress. Of particular interest is that spores are highly sensitive to their microenvironments before they produce germ tubes and this will clearly need to be taken into account in order to understand the intimate and highly dynamic interplay of rapid signalling processes that occur within the infection court to determine the establishment of the pathogen-host cell interaction. This study highlights the utility of single cell fluorescence reporters to gain a deeper understanding of these processes.

## 3 Methods

The *A. fumigatus* strains used were GCaMP5^*ΔakuB*^ (*ΔakuB*^KU80^*;AngpdA*^*P*^*-GCaMP5-ptrA*) and GCaMP6^*ΔakuB*^ (*ΔakuB*^KU80^*;AngpdA*^*P*^*-GCaMP5-ptrA*), which were generated by transforming the parental isolate *ΔakuB*^KU80^ (da Silva Ferreira et al., 2006) with the plasmids pSK379-GCaMP5 and pSK739-GCaMP6 according to the protocol described in Szewczyk *et al*., 2006. These plasmids target the insertion of the expression cassette containing GCaMPs under the control of the *A. nidulans gpdA* promoter (*AngpdA*) at the 3’-flanking region of the *A. fumigatus* histone 2A locus (*his2A*) (Wagener et al., 2008). The *A. oryzae* pyrithiamine resistance marker (ptrA) was used for selection in fungal cells (0.5 ug/ml pyrithiamine). Transformants were verified for the presence, site of genomic integration and single copy number of GCaMP5 and GCaMP6 using PCR (using the oligonucleotides QC_GCaMP_1 GTCAGAGCTATAGGTCGG and QC_GCaMP_2 CTTGAAGTCGATGCCCTT) and Southern blotting using GCaMP- and *his2A*-specific probes (Supplementary Fig. 1). For Southern blotting analysis, genomic DNA was digested with the restriction enzyme EcoRI. Relative to the parental isolate *ΔakuB*^KU80^, no growth alterations due to the *AngpdA*-driven expression of the reporters GCaMP5 and GCaMP6 were observed upon growth onto solid media Aspergillus Complete media (ACM) (Supplementary Fig. 2). Plasmids and strains generated were deposited into the collection of the Manchester Fungal Infection Group.

For preparation of the experiments, strains were harvested using sterile H_2_O from cultures grown on solid ACM for 5 days at 37°C (Pontecorvo et al., 1953). Spore suspensions were filtered using Miracloth (Calbiochem), centrifuged for 10 min at 4000 rpm and washed twice with sterile H_2_O. Spores were enumerated using a haemocytometer and resuspended to the desired concentration in sterile H_2_O.

The *A. fumigatus* strains GCaMP5^*ΔakuB*^ and GCaMP6^*ΔakuB*^ were cultured in Aspergillus Minimal media (AMM) (Pontecorvo et al., 1953). Dormant conidia were transferred to a Cellasic microfluidic culture chamber (Merck) and incubated at 25°C. Conidia and subsequently conidial germlings were immobilized by between silicone and glass whilst being kept in focus and exposed to AMM supplemented with 200 mM CaCl_2_ and applied by continuous perfusion at 0.5-1 psi flow rates for the duration of the time course.

## Supplementary Material

Supplementary Materials

Supplementary Materials

## Figures and Tables

**Movie. 1:**
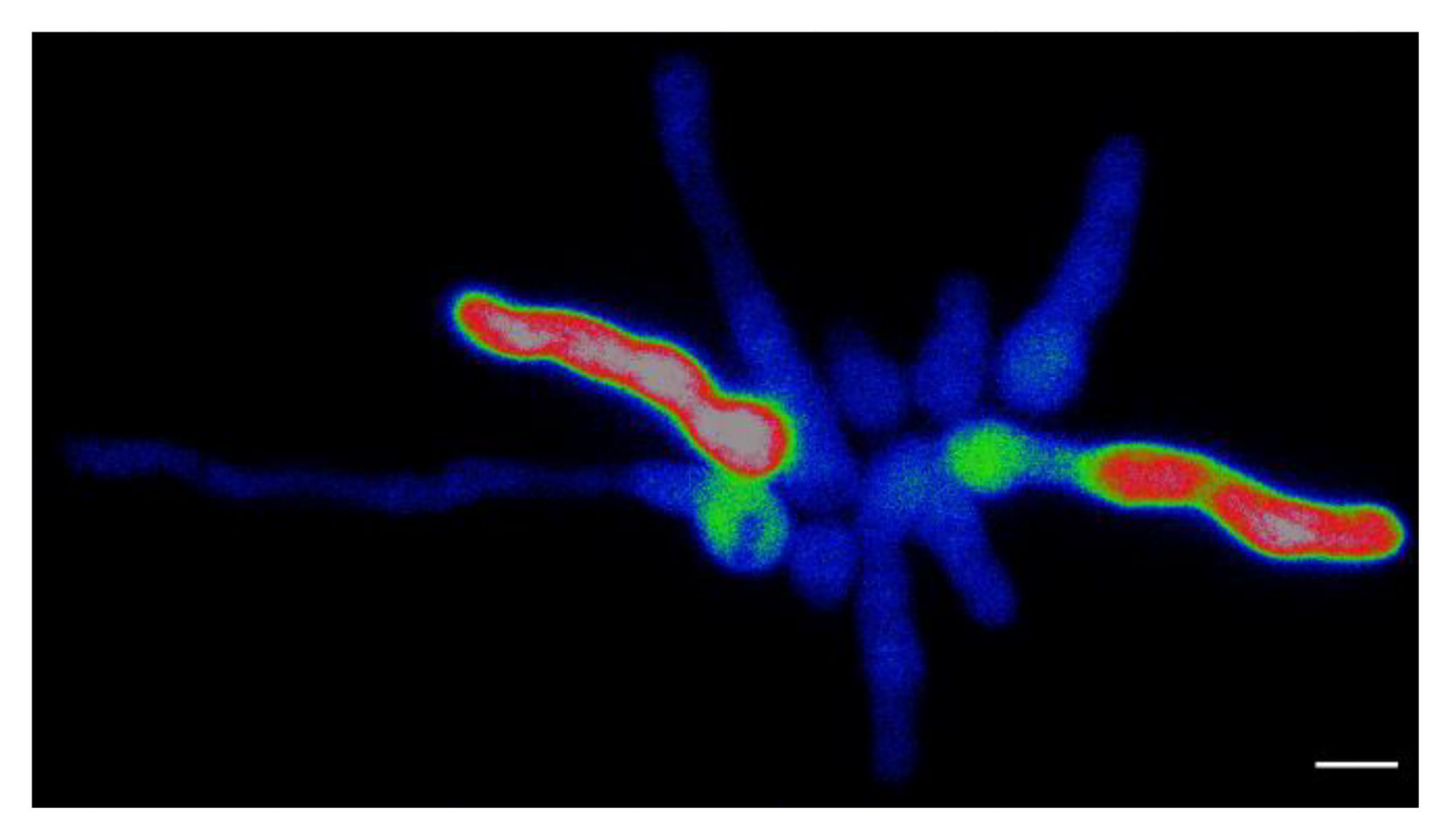
Calcium imaging reveals enhanced [Ca^2+^]_c_ dynamics and heterogeneity in the timing of Ca^2+^ responses between germlings of *A. fumigatus* expressing GCaMP5. Germination and incubation were carried out in a microfluidic chamber for 18 h at 25°C with a flow rate of AMM of 0.5-1 psi. The germlings were then exposed to high Ca^2+^ stress (AMM containing 200 mM CaCl_2_) that was added at the beginning of a 300 sec analytical time course experiment. The high Ca^2+^ stress was continuously maintained over the course of the movie. Time resolution = 1 fps (frame per second). Scale bar = 5 μm.

**Movie. 2:**
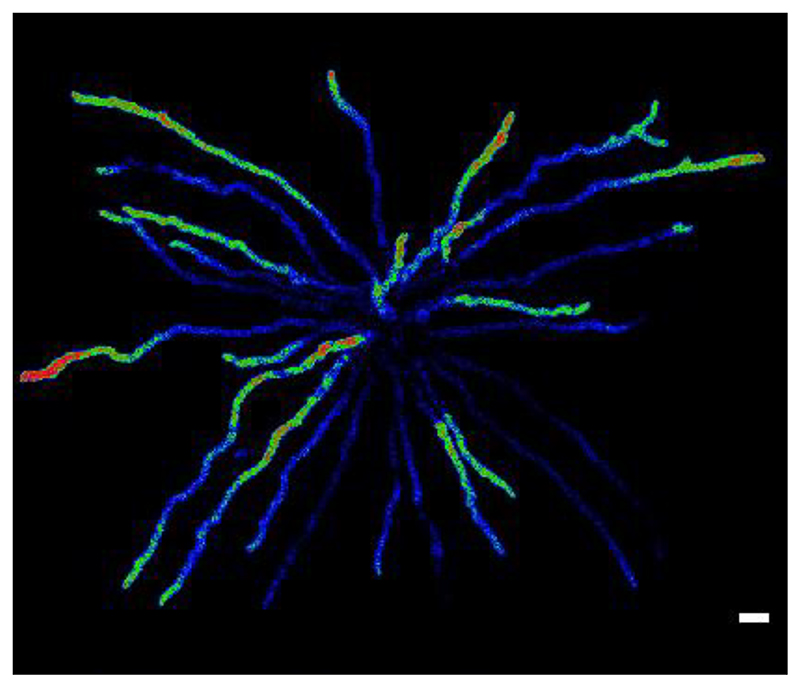
Rapid and increased [Ca^2+^]_c_ dynamics are seen in *A. fumigatus* hyphae expressing GCaMP6. Germination and incubation were carried out in a microfluidic chamber for 21 h at 25°C with a flow rate of AMM of 0.5-1 psi. *A. fumigatus* cells were then exposed to high Ca^2+^ stress (AMM containing 200 mM CaCl_2_) that was added at the beginning of a 20 min analytical time course experiment. Furthermore, during the course of this experiment an extra stimulus with another 200 mM CaCl_2_ at the 15 min time point was perfused into the chamber. Time resolution = 33.5 fpm (frame per minute). Scale bar = 10 μm.

**Movie. 3:**
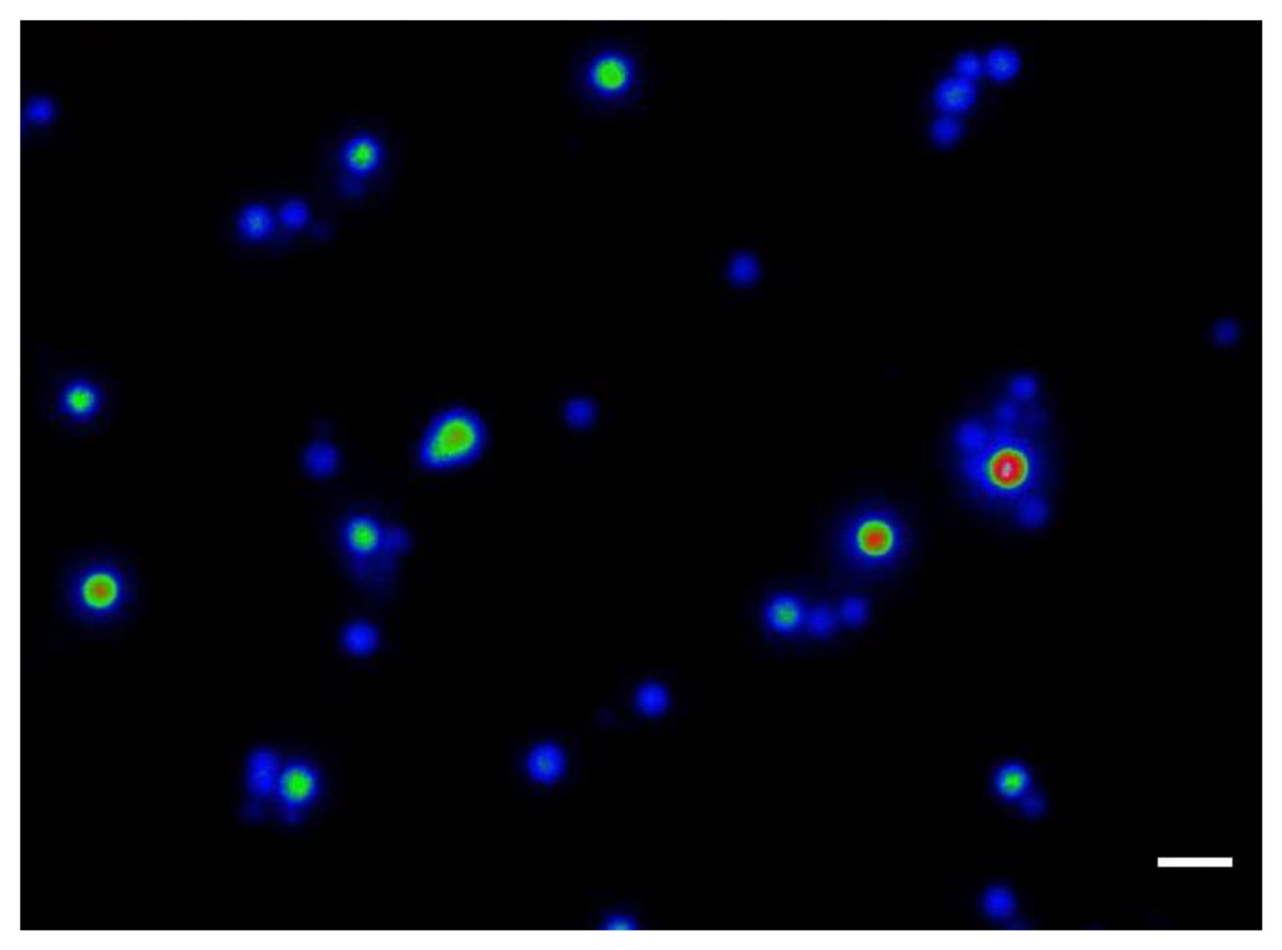
Ungerminated, swollen and nascent pre-polarised hyphae of *A. fumigatus* expressing GCaMP6 undergo Ca^2+^ signalling. Spores were pre-incubated in a microfluidic chamber for 16 h at 25°C with a flow rate of AMM of 0.5-1 psi. Individual fungal cells were then exposed to high Ca^2+^ stress (200 mM CaCl_2_) that was applied after 1 min from the beginning of a 10 min analytical time course. The single or multiple [Ca^2+^]_c_ transients and the [Ca^2+^]_c_ responses exhibited by individual fungal cells show marked heterogeneity with regard to the timing of these transients relative to others in the subpopulation of cells. Time resolution = 1 fps (frame per second). Scale bar = 10 μm.

## Highlights

- Calcium signalling plays a fundamental role in rapid fungal intracellular signalling
- Previous results of imaging fungal cytosolic free calcium ([Ca^2+^]_c_) have been inconsistent in fungi
- Recent genetically encoded GCaMP fluorescent probes provide excellent imaging of rapid [Ca^2+^]_c_ dynamics at the subcellular level in fungi
- Exposing conidia or conidial germlings to high external Ca^2+^ induces very dynamic [Ca^2+^]_c_ changes with localized [Ca^2+^]_c_ transients and waves
- Considerable heterogeneity in the timing of [Ca^2+^]_c_ responses of different ungerminated conidia or conidial germlings within a cell population is observed
