# Supplementary material for: Live-cell imaging of rapid calcium dynamics using fluorescent, genetically-encoded GCaMP probes with Aspergillus fumigatus
Source: Fungal Genet Biol. Author manuscript; Available in PMC 2025 Jul 20. (PMC7617832; doi:10.1016/j.fgb.2020.103470)
Supplement: Supplementary Materials [file EMS206533-supplement-Supplementary_Materials.zip › 1-s2.0-S1087184520301614-mmc4.docx]

**SUPPLEMENTARY**

**SUPPLEMENTARY DATA**

**Plasmid construction.** The cloning vehicle for targeted insertion of the CGaMP reporter into the *A. fumigatus* genome was pSK379, an integrative plasmid which targets the insertion of the expression cassette containing GCaMP under the control of the *A. nidulans* *gpdA* promoter at the 3'-flanking region of the *A. fumigatus* *his2A* gene locus (Wagener *et al.*, 2008). The ampicillin resistance (bla) and the *A. oryzae* pyrithiamine resistance marker (ptrA) markers were used for selection and maintenance in bacterial and fungal cells respectively. To generate the plasmids pSK379-GCaMP5 and pSK739-GCaMP6, the GCamp5 and GCaMP6 genes were amplified using the oligonucleotides GCaMP_1 CCATGGGTTCTCATCATCATCATC and GCaMP_2 CCTGAATGTTGAGTGGAATGATGG from the plasmids pSK3042 (Kind gift of Seogchan Kang, Department of Plant Pathology and Environmental Microbiology, The Pennsylvania State University) and pGP-CMV-GCaMP6s (Addgene), respectively. Following this step, the GCaMP cassettes were blunt-ended, phosphorylated and ligated into PmeI-digested and dephosphorylated pSK379. The resulting plasmids will express GCaMP5 and GCaMP6 following a sequence encoding the promoter region (from -433 to -1 from the ATG) of the constitutively expressed *Aspergillus nidulans* glyceraldehyde-3-phosphate dehydrogenase gene (ANIA_08041) and will also contain a 1997 bp sequence identical to the 3'-flanking region of the *A. fumigatus* *his2A* gene (AFUA_3G05360) for targeting into *A. fumigatus* genome. Both plasmids were fully sequenced and deposited into the collection of the Manchester Fungal Infection Group.

**GCamp insert sequences.** In red is indicated the half sequence of the PmeI site after the cloning (cut in position 2715 bp in original pSK379 plasmid), while highlighted in yellow and green are the sequences for the oligonucleotides QC_GCaMP_1 and QC_GCaMP_2 used for PCR verification.

pSK379-GCaMP5

PmeI

CAGACATCACCGTTTCCATGGGTTCTCATCATCATCATCATCATGGTATGGCTAGCATGACTGGTGGACAGCAAATGGGTCGGGATCTGTACGACGATGACGATAAGGATCTCGCCACCATGGTCGACTCATCACGTCGTAAGTGGAATAAGACAGGTCACGCAGTCAGAGCTATAGGTCGGCTGAGCTCACTCGAGAACGTCTATATCAAGGCCGACAAGCAGAAGAACGGCATCAAGGCGAACTTCAAGATCCGCCACAACATCGAGGACGGCGGCGTGCAGCTCGCCTACCACTACCAGCAGAACACCCCCATCGGCGACGGCCCCGTGCTGCTGCCCGACAACCACTACCTGAGCGTGCAGTCCAAACTTTCGAAAGACCCCAACGAGAAGCGCGATCACATGGTCCTGCTGGAGTTCGTGACCGCCGCCGGGATCACTCTCGGCATGGACGAGCTGTACAAGGGCGGTACCGGAGGGAGCATGGTGAGCAAGGGCGAGGAGCTGTTCACCGGGGTGGTGCCCATCCTGGTCGAGCTGGACGGCGACGTAAACGGCCACAAGTTCAGCGTGTCCGGCGAGGGTGAGGGCGATGCCACCTACGGCAAGCTGACCCTGAAGTTCATCTGCACCACCGGCAAGCTGCCCGTGCCCTGGCCCACCCTCGTGACCACCCTGACCTACGGCGTGCAGTGCTTCAGCCGCTACCCCGACCACATGAAGCAGCACGACTTCTTCAAGTCCGCCATGCCCGAAGGCTACATCCAGGAGCGCACCATCTTCTTCAAGGACGACGGCAACTACAAGACCCGCGCCGAGGTGAAGTTCGAGGGCGACACCCTGGTGAACCGCATCGAGCTGAAGGGCATCGACTTCAAGGAGGACGGCAACATCCTGGGGCACAAGCTGGAGTACAACCTGCCGGACCAACTGACTGAAGAGCAGATCGCAGAATTTAAAGAGGCTTTCTCCCTATTTGACAAGGACGGGGATGGGACAATAACAACCAAGGAGCTGGGGACGGTGATGCGGTCTCTGGGGCAGAACCCCACAGAAGCAGAGCTGCAGGACATGATCAATGAAGTAGATGCCGACGGTGACGGCACAATCGACTTCCCTGAGTTCCTGACAATGATGGCAAGAAAAATGAAATACACAGACAGTGAAGAAGAAATTAGAGAAGCGTTCCGTGTGTTTGATAAGGATGGCAATGGCTACATCAGTGCAGCAGAGCTTCGCCACGTGATGACAAACCTTGGAGAGAAGTTAACAGATGAAGAGGTTGATGAAATGATCAGGGAAGCAGACATCGATGGGGATGGTCAGGTAAACTACGAAGAGTTTGTACAAATGATGACAGCGAAGTAAGCATGCCATCATTCCACTCAACATTCAGGAAACGCCATGTCTA

PmeI

pSK379-GCaMP6

PmeI

CAGACATCACCGTTTCCATGGGTTCTCATCATCATCATCATCATGGTATGGCTAGCATGACTGGTGGACAGCAAATGGGTCGGGATCTGTACGACGATGACGATAAGGATCTCGCCACCATGGTCGACTCATCACGTCGTAAGTGGAATAAGACAGGTCACGCAGTCAGAGCTATAGGTCGGCTGAGCTCACTCGAGAACGTCTATATCAAGGCCGACAAGCAGAAGAACGGCATCAAGGCGAACTTCCACATCCGCCACAACATCGAGGACGGCGGCGTGCAGCTCGCCTACCACTACCAGCAGAACACCCCCATCGGCGACGGCCCCGTGCTGCTGCCCGACAACCACTACCTGAGCGTGCAGTCCAAACTTTCGAAAGACCCCAACGAGAAGCGCGATCACATGGTCCTGCTGGAGTTCGTGACCGCCGCCGGGATCACTCTCGGCATGGACGAGCTGTACAAGGGCGGTACCGGAGGGAGCATGGTGAGCAAGGGCGAGGAGCTGTTCACCGGGGTGGTGCCCATCCTGGTCGAGCTGGACGGCGACGTAAACGGCCACAAGTTCAGCGTGTCCGGCGAGGGTGAGGGCGATGCCACCTACGGCAAGCTGACCCTGAAGTTCATCTGCACCACCGGCAAGCTGCCCGTGCCCTGGCCCACCCTCGTGACCACCCTGACCTACGGCGTGCAGTGCTTCAGCCGCTACCCCGACCACATGAAGCAGCACGACTTCTTCAAGTCCGCCATGCCCGAAGGCTACATCCAGGAGCGCACCATCTTCTTCAAGGACGACGGCAACTACAAGACCCGCGCCGAGGTGAAGTTCGAGGGCGACACCCTGGTGAACCGCATCGAGCTGAAGGGCATCGACTTCAAGGAGGACGGCAACATCCTGGGGCACAAGCTGGAGTACAACCTGCCGGACCAACTGACTGAAGAGCAGATCGCAGAATTTAAAGAGGCTTTCTCCCTATTTGACAAGGACGGGGATGGGACAATAACAACCAAGGAGCTGGGGACGGTGATGCGGTCTCTGGGGCAGAACCCCACAGAAGCAGAGCTGCAGGACATGATCAATGAAGTAGATGCCGACGGTGACGGCACAATCGACTTCCCTGAGTTCCTGACAATGATGGCAAGAAAAATGAAATACAGGGACACGGAAGAAGAAATTAGAGAAGCGTTCGGTGTGTTTGATAAGGATGGCAATGGCTACATCAGTGCAGCAGAGCTTCGCCACGTGATGACAAACCTTGGAGAGAAGTTAACAGATGAAGAGGTTGATGAAATGATCAGGGAAGCAGACATCGATGGGGATGGTCAGGTAAACTACGAAGAGTTTGTACAAATGATGACAGCGAAGTGAGCAAACGCCATGTCTA

PmeI

**SUPPLEMENTARY FIGURES**

**Supplementary Figure 1:** Single, targeted integration of the GCaMP expression constructs in the GCaMP^ΔakuB^ strains was verified by Southern blotting. **(A)** The Southern blot strategy hybridised *EcoRI*-digested genomic DNA with GCaMP- or *his2A*-specific probes, amplified with the oligonucleotide pairs GCaMPF (GTCAGAGCTATAGGTCGG) – GCaMPR (CTTGAAGTCGATGCCCTT) and LucSBF (GTAACTACGCTCAACGTGTT) – LucSBR (GAAAGCTGTCGGTATCATTC) respectively. For the GCaMP^ΔakuB^ strains a 3626 bp region of the native chromosomal locus will become interrupted by insertion, via a single cross-over event, of the 8770 bp pSK379-GaMP plasmid, itself bearing two EcoRI sites. The placement of this plasmid within the insertion site will introduce 2 new EcoRI sites. For the GCaMP-specific probe, no band is expected for the parental isolate, whereas a single band of a maximum size of 4674 bp (dependent upon the precise site of integration) is expected for the reporter strains. For the his2A-specific probe, a band of 3626 bp is expected for the parental isolate, whereas a single band of a maximum size of 7481 bp (dependent upon the precise site of integration) is expected for the reporter strains. **(B)** Verification for the GCaMP^ΔakuB^ strains.

**Supplementary Figure 2:** Comparative growth of GCaMP^ΔakuB^ strains and respective parental isolate *ΔakuB*^KU80^ as serial dilutions of 10^5^-10^2^ spores in ACM for 48 hrs.

**Supplementary Figure 3:** Comparative analysis of the Fluorescence Intensity (F.I.) versus Time for the germlings and spores shown in Movies 1 (top graph in A) and 3 (bottom graph in B), respectively. Each line corresponds to an individual cell within the group of ten germlings shown in Movie 1 and 45 spores in Movie 3.

**REFERENCES**

Wagener J, Echtenacher B, Rohde M, Kotz A, Krappmann S, Heesemann J & Ebel F (2008) The putative alpha-1,2-mannosyltransferase AfMnt1 of the opportunistic fungal pathogen *Aspergillus fumigatus* is required for cell wall stability and full virulence. *Eukaryot Cell* **7**: 1661-1673.
