# Supplementary material for: Live-cell imaging of rapid calcium dynamics using fluorescent, genetically-encoded GCaMP probes with Aspergillus fumigatus
Source: Fungal Genet Biol. Author manuscript; Available in PMC 2025 Jul 20. (PMC7617832; doi:10.1016/j.fgb.2020.103470)
Supplement: Supplementary Materials [file EMS206533-supplement-Supplementary_Materials.pdf]

Movie 1

Click here to access/download

Video

Movie 1- Germ tubes expressing GCaMP5 and exposed to 200 mM CaCl2.pptx

Movie 2

Click here to access/download

Video

Movie 2 - Germ tubes expressing GCaMP6 and exposed to 200 mM CaCl2.pptx

Movie 3

Click here to access/download
Video
Movie 3 - Spores exposed to 200 mM CaCl2.pptx

Movie 1 avi file

Click here to access/download
Video
MOV1.avi

Movie 2 avi file

Click here to access/download
Video
MOV2.avi

Movie 3 avi file

Click here to access/download
Video
MOV3.avi

Click here to access/download
Supporting File
Supplementary_final.docx
